# Germline heterozygous SH2B3‐mutations and (idiopathic) erythrocytosis: Detection of a previously undescribed mutation

**DOI:** 10.1002/jha2.800

**Published:** 2023-10-27

**Authors:** Gaël Vermeersch, Timothy Devos, Helena Devos, Frédéric Lambert, Bruce Poppe, Sam Van Hecke

**Affiliations:** ^1^ Department of Hematology University Hospitals Leuven Leuven Belgium; ^2^ Department of Hematology AZ Damiaan Ostend Belgium; ^3^ Department of Microbiology and Immunology Laboratory of Molecular Immunology (Rega Institute), KU Leuven Leuven Belgium; ^4^ Department of Laboratory Hematology AZ Sint‐Jan Brugge‐Oostende AV Bruges Belgium; ^5^ Center for Human Genetic, Molecular Hemato‐Oncology Unit UniLab Liège, Centre Hospitalier Universitaire de Liège Liège Belgium; ^6^ Center for Medical Genetics Ghent University Hospital Ghent Belgium

**Keywords:** erythrocytosis, idiopathic erythrocytosis, myeloid function and development, myeloproliferative disorder

## Abstract

Erythrocytosis or polycythemia refers to a true or apparent increase in hemoglobin or hematocrit. When no etiology of erythrocytosis is identified, people are diagnosed with “idiopathic erythrocytosis” (IE). The identification of new contributing genes has recently improved the diagnostic workup of IE. As such mutations within the *SH2B3* gene, which codes for the LNK protein and negatively regulates the JAK‐STAT pathway, have been identified in cases diagnosed as IE. This reports describes the presence of a previously undescribed germline *SH2B3* variant p.(Thr335ArgfsTer4) within IE and emphasizes the advantages of gene panel sequencing as second step in the diagnostic work‐up.

1

Erythrocytosis or polycythemia refers to a true or apparent increase in hemoglobin or hematocrit. According to the 2016 World Health Organization (WHO)‐classification a hemoglobin/hematocrit level of ≥165 g/L/≥49% in men and ≥160 g/L/≥48% in women has been determined to define myeloproliferative disease. Approximately 7.6% of males and 0.4% of females reach current diagnostic criteria [1, 2, 3]. Relative polycythemia refers to a decrease in plasma volume while absolute polycythemia is caused by an increase of red blood cell count. Absolute erythrocytosis can be categorized as primary or secondary. “Primary” is due to mutation(s) in progenitor cells, “secondary” may be caused by hypoxia, erythropoietin (EPO)‐producing neoplasms or medication such as testosterone. Most primary cases are acquired, by example polycythemia vera (PV) in which approximately 95% of cases carry the *JAK2* p.Val617Phe mutation. The Janus kinase‐signal transducer and activator of transcription signaling pathway (JAK‐STAT) is important in erythroid proliferation in response to EPO signaling. Inherited mutations may cause erythrocytosis by impacting hypoxia sensing, JAK‐STAT signaling or epigenetic mechanisms [1, 3]. Identification of new causal/contributing genes improves the diagnostic workup of “idiopathic erythrocytosis” (IE). We report a new germline *SH2B3* variant (p.Thr335ArgfsTer4) for the first time in erythrocytosis. The SH2B adaptor protein 3 gene *(SH2B3)* codes for the lymphocyte adaptor protein (LNK), which negatively regulates the JAK‐STAT pathway [4, 5, 6, 7].

A 31‐years old Caucasian male was referred because of polycythemia and mild thrombocytopenia (pseudothrombopenia excluded). He experienced no complaints of vasomotor symptoms, pruritus or B‐symptoms. Blood oxygen levels were normal. He was an active smoker; there was no alcohol abuse or use of medication. There were hematological disorders known within family history. His medical history included autism spectrum disorder; there were no thrombo‐embolic events. Clinical investigation was unremarkable, body‐mass index was 24.6 kg/m^2^. Biochemical analysis (Table 1) showed a hemoglobin and hematocrit value of respectively 242 g/L (reference range (RR):135‐175 g/L) and 70.9% (RR:41.0‐53.0%). Thrombocytes were 72 × 10^9/L (RR:150‐450 × 10^9/L). Levels of testosterone and EPO resulted normal. Venous p50 was 25.1 mmHg. Thoraco‐abdominal computed tomography and karyotyping (46,XY [10]) resulted normal. Bone marrow biopsy showed no signs of myeloproliferative neoplasms (MPN) according to current WHO‐criteria. *JAK2* p.Val617Phe, exon12 and other variants were absent. No mutations in the *EPOR*, *VHL*, *PHD2/EGLN1* and *HIF2A/EPAS1* genes were found. Bone marrow molecular analysis (Supplemental Data) revealed following variants: *SH2B3* (NM_005475.3, c.1004_1005del, p.Thr335ArgfsTer4; Variant Allele Frequency (VAF) 51%)(American College of Medical Genetics (ACMG) classification as “likely pathogenic”), *JAK3* (NM_000215.4, p.Arg540His, VAF 49%)(ACMG:“likely benign”), *RELN* (NM_005045.4, p.Pro65Leu, VAF 51%)(ACMG:“uncertain significance”). The variants of *SH2B3*, *JAK3* and *RELN* were confirmed on buccal swab DNA (VAF respectively 52%, 49% and 50%) and hair bulb DNA (VAF 49%, 49% and 53%). Genetic analysis of maternal peripheral blood (hemoglobin 147 g/L, hematocrit 44%, menopausal, 55 years old) confirmed heterozygous carriership of mutated *SH2B3*. Paternal genetic analysis was not performed as contact was lost since several years.

**TABLE 1:** Blood results at time of presentation.

Blood tests at presentation			
Blood test (general)	Blood test (specific)	Value	Reference range
Hematology	Leukocytes	7.3	4.0–10.0 × 10^9/L
	Hemoglobin	242	135–175 g/L
	Hematocrit (%)	70.9	41.0%–53.0%
	Reticulocytes	114.9	30.0–100.0 × 10^9/L
	Thrombocytes	72 (EDTA)	150–450 × 10^9/L
		56 (citrate)	
Coagulation	INR	1.48	0.90–1.20
	aPTT	41.1	23.7–34.9 s
Inflammation	C‐reactive protein	1.7	<5.0 mg/L
Iron	Iron	163	59–158 μg/dL
	Transferrin saturation (%)	44	16%–45%
	Ferritin	96	96 mg/L
Kidney	Creatinine	0.78	<1.20 mg/dL
Liver tests	AST	33	<37 mg/dL
	ALT	41	<41 mg/dL
	GGT	44	<61 mg/dL
	Total bilirubin	0.97	<1.10 mg/dL
Electrophoresis	Albumin	48.9	40.2–47.6 g/L
	Alpha‐1‐globulin	3.1	2.1–3.5 g/L
	Alpha‐2‐globulin	6.3	5.1–8.5 g/L
	Beta‐globulin	6.7	6.0–9.4 g/L
	Gamma‐globulin	16.2	16.2 g/L
Immunoglobulins	IgG	15.57	7.00–16.00 g/L
	IgA	1.53	0.70–4.00 g/L
	IgM	1.19	0.40–2.30 g/L
Hormones	DHEAS	397.94	160–449 μg/dL
	Testosterone	18.3	8.64–29 nmol/L
	Testosterone free	70.5	47–224 pg/mL
	Sex‐hormone binding globulin	58	18–54 nmol/L
	Erythropoietin	14.4 U/L	4.3–29.0 U/L
Infectious serology	HBV surface antigen	Negative	Negative
	HBV core antibodies	Negative	Negative
	HBV surface antibodies	Positive (74 U/L)	/
	Hepatitis C antibodies	Negative	Negative
	HIV	Negative	Negative
Arterial blood sample	pH	7.44	7.35–7.45
	pCO2	34.8	35.0–48.0 mmHg
	pO2	Not calculable	83–108 mmHg
	Saturation (%)	99	
	Carboxyhemoglobin (%)	3.6	0.5%–1.5% (nonsmoker)
			4.0%–9.0% (smoker)
Venous blood sample	pH	7.33	7.32–7.42
	pCO2	51.9	41.0–51.0 mmHg
	pO2	27.6	25.0–40.0 mmHg
	Saturation (%)	51	45.0%–70.0%
	Carboxyhemoglobin (%)	3.5	0.5%–1.5%
	p50	25.1	24–32 mmHg

Abbreviations: ALT, alanine aminotransferase; aPTT, activated partial thromboplastin time; AST, aspartate aminotransferase; DHEAS, dehydroepiandrosterone sulfate; GGT, gamma‐glutamyl transferase; HBV, hepatitis B virus; HIV, human immunodeficiency virus; IgA, immunoglobulin A; IgG, immunoglobulin G; IgM, immunoglobulin M; INR, international normalized ratio.

The *SH2B3* gene codes for the LNK protein (figure 1) and is predominantly expressed in hematopoietic cells. LNK is member of the SH2‐domain‐containing adaptor family of proteins which exist out of a N‐terminal dimerization domain, a central pleckstrin homology domain (PH) and C‐terminal Src homology 2 (SH2) domain. The PH‐domain interacts with phosphatidylinositol lipids in the cellular membrane and assists localization of proteins such as SH2. SH2 modulates hematopoiesis through direct interaction with JAK2 or the EPO‐receptor (pY454) [4, 8, 9]. Interaction with JAK2 (pY813) occurs through a binding pocket formed by residues Arg343, Arg364, Ser366, Arg369, His385, and Arg387 [8]. Mutated *SH2B3* is described in MPN and nonmalignant diseases as IE. IE compromises a heterogenous group of disorders without evidence for MPN. MPNs are subdivided as *BCR::ABL*‐positive or ‐negative, respectively indicating the presence/absence of a reciprocal translocation between chromosome 9 and 22. Typical chronic myeloid leukemia is *BCR::ABL*‐positive. *BCR::ABL*‐negative MPN are generally classified as PV, essential thrombocythemia and primary myelofibrosis. Somatic mutations of *SH2B3* occur in 5%–7% of MPN patients and are associated with leukemic transformation [5, 10]. Most *SH2B3* mutations occur in exon 2 (residues Glu208‐Asp234), which codes for the PH‐domain, residue 208 is considered as a mutational hotspot (p.Glu208Gln) [7, 11].

**FIGURE 1:**
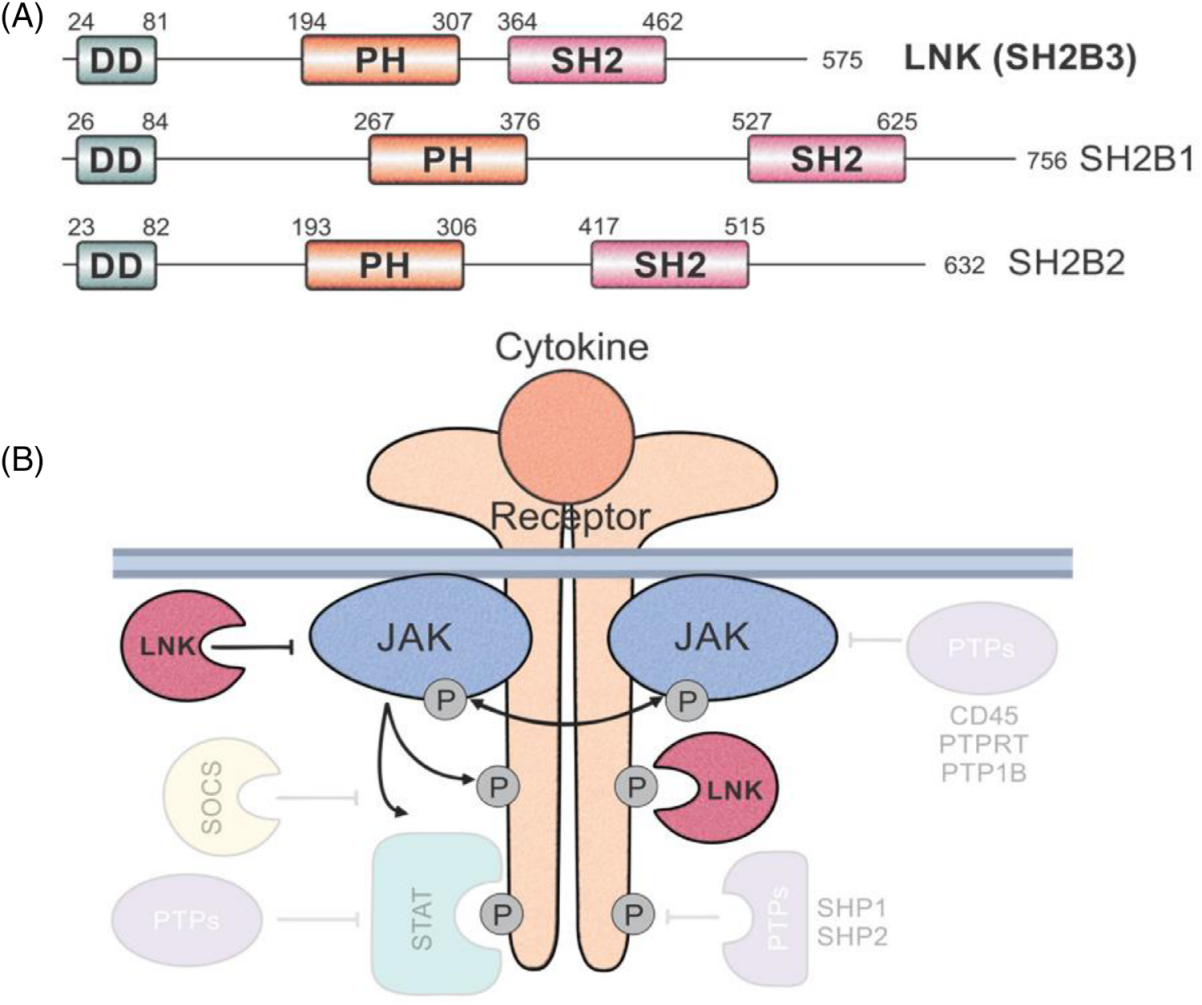
Graphical representation of SH2B3 and the interaction with JAK‐STAT. (A) The SH2B adaptor protein 3 gene, located on chromosome 12, codes for the lymphocyte adaptor protein (LNK), also known as SH2B3. This adaptor family also contains SH2B1 and SH2B3. The Src homology 2 (SH2) domain‐containing adaptor family shows the same protein architecture, existing out of an N‐terminal dimerization domain (DD), pleckstrin homology domain (PH) and a C‐terminal SH2 domain. (B) LNK may directly inhibit the Janus Kinase‐signal transducer and activator of transcription (JAK‐STAT) signaling pathway. Other interacting proteins (semi‐transparent color) such as phosphatases and suppressor of cytokine signaling (SOCS) are not discussed in this article. (Figure extracted from: Morris R, Butler L, Perkins A, Kershaw NJ, Babon JJ. The role of lnk (Sh2b3) in the regulation of jak‐stat signaling in haematopoiesis. Pharmaceuticals 2022;15. https://doi.org/10.3390/ph15010024).

This is the first report describing the germline *SH2B3* mutation p.(Thr335ArgfsTer4) in IE. Residue 335 is coded by exon 5. The Varsome database classifies this variant as “likely pathogenic,” it is not found in the COSMIC and Human Gene Mutation Database. As in our case most *SH2B3* mutations appear to be heterozygous, whether these contribute through haploinsufficiency or a dominant‐negative effect is unknown [5]. The lack of in vitro proof of concept of this variant could be considered as a limitation of this report. It is not explained why the mother has no signs of erythrocytosis, a synergistic effect of the patient's active smoking or other genes cannot be excluded. The patient carries mutated *JAK3* and *RELN* with similar VAF. JAK3 belongs to the JAK‐family of proteins and is mainly restricted to the hematopoietic lineage. LNK interacts with JAK3 and may serve as a scaffold enabling JAK3 autophosphorylation in the absence of a cytokine receptor. LNK mutations as Glu208Gln result in augmented phosphorylation [12]. Mutated *RELN*, which encodes for proteins functioning in cellular interaction, is reported as megakaryocyte‐unique somatic mutation in patients with MPN and may predispose to megakaryocytic clustering. No clustering was present in the bone marrow of this patient. Mutated *RELN* is reported in autism spectrum disorder, as in our case, and in epilepsy [13, 14]. The potential role of these mutations in IE is unclear.

By following treatment guidelines we initiated low dose acetylsalicylic acid and performed venesection. An arbitrary hematocrit target of ≤55% is used; ≤45% is considered in patients with history of erythrocytosis‐related thrombosis. Smoking cessation was advised [15]. Hematocrit declined to ≤55% after four venesections performed within 7 weeks, thrombocyte count normalized (Supplemental Figure 1).

Although no specific phenotype is associated with mutated *SH2B3* there is growing interest in its role in myeloproliferative disorders. This report supports gene panel sequencing as a second step in the diagnostic work‐up of erythrocytosis as it is able to open new diagnostic opportunities of potential germline associated conditions [7]. Identifying the genomic landscape in erythrocytosis will hopefully result in better risk‐stratification and treatment optimization.

## AUTHOR CONTRIBUTIONS

The manuscript was written by G. Vermeersch and S. Van Hecke. The manuscript was critically revised by T. Devos, H. Devos, F. Lambert and B. Poppe. Genetic analyses were performed by the “ Department of Laboratory Hematology, AZ Sint‐Jan Brugge‐Oostende AV,” “Center for Human Genetic, UniLab Liège, Centre Hospitalier Universitaire de Liège,” and “Center for Medical Genetics, Ghent University Hospital,” under supervision of respectively H. Devos, F. Lambert and B. Poppe.

## CONFLICT OF INTEREST STATEMENT

The authors declare they have no conflict of interest.

## ETHICS STATEMENT

The authors have confirmed ethical approval statement is not needed for this submission.

## PATIENT CONSENT STATEMENT

The authors have confirmed consent statement was obtained from the patient and mother.

## PERMISSION TO REPRODUCE MATERIAL FROM OTHER SOURCES

The authors have confirmed permission was obtained.

## CLINICAL TRIAL REGISTRATION

The authors have confirmed clinical trial registration is not needed for this submission.

## Supporting information

Figure S1: Overview of biochemical evolution during follow‐up. (A) Graphical representation of hematocrit and thrombocyte count during follow up. Venesection was not performed on week 15 and 22. (B) Numeric values of hematocrit, hemoglobin concentration, and thrombocyte count during follow‐up.

## Data Availability

The data that support the findings of this study are available upon request to the corresponding author. The data are not publicly available due to privacy or ethical restrictions.
