# Supplementary material for: Germline heterozygous SH2B3‐mutations and (idiopathic) erythrocytosis: Detection of a previously undescribed mutation
Source: EJHaem. 2023 Oct 27;4(4):1143–7. doi: 10.1002/jha2.800 (PMC10660405; doi:10.1002/jha2.800)

## Slide 1
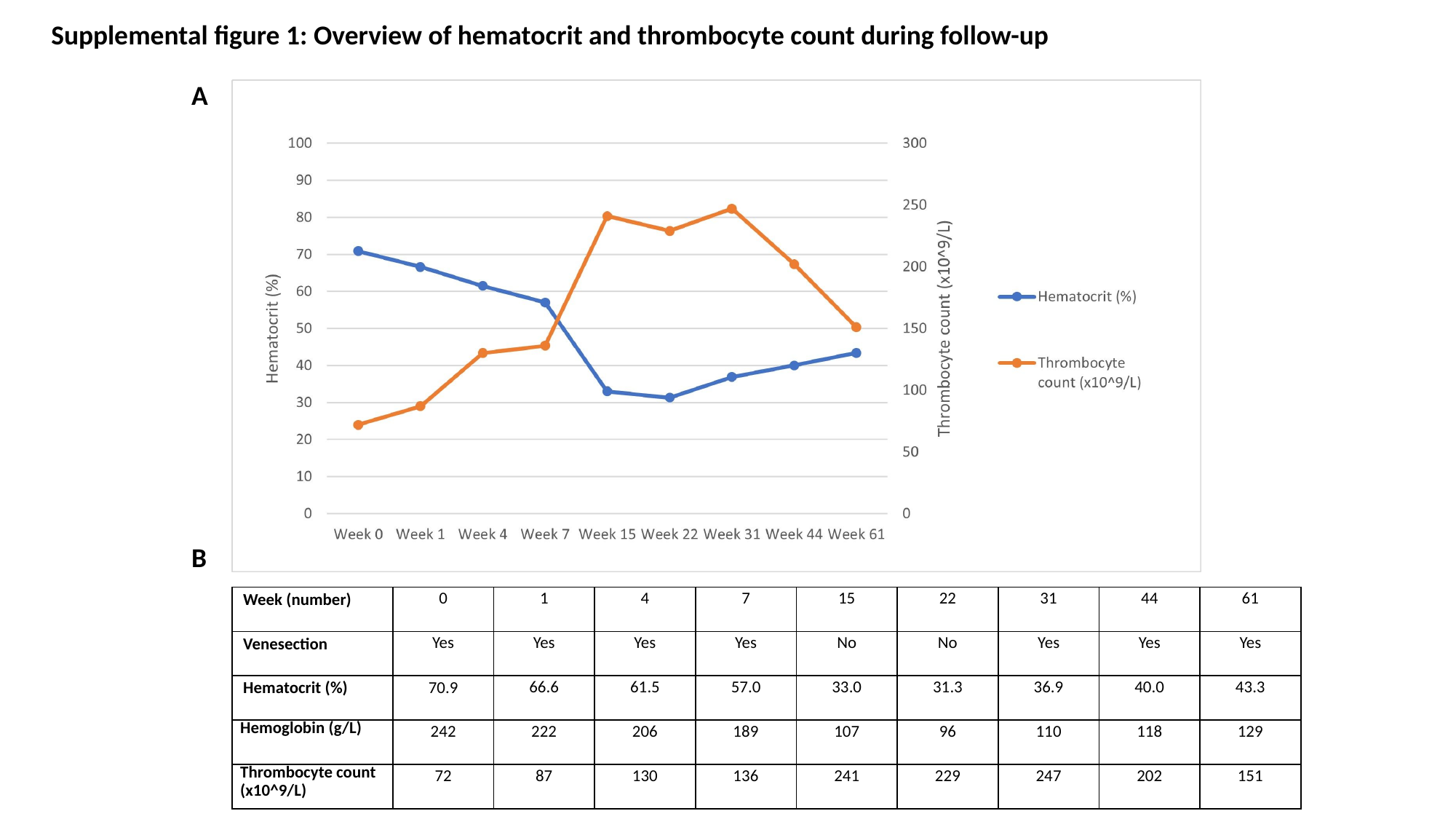

Supplemental figure 1: Overview of hematocrit and thrombocyte count during follow-up
A
B
| Week (number) | 0 | 1 | 4 | 7 | 15 | 22 | 31 | 44 | 61 |
| --- | --- | --- | --- | --- | --- | --- | --- | --- | --- |
| Venesection | Yes | Yes | Yes | Yes | No | No | Yes | Yes | Yes |
| Hematocrit (%) | 70.9 | 66.6 | 61.5 | 57.0 | 33.0 | 31.3 | 36.9 | 40.0 | 43.3 |
| Hemoglobin (g/L) | 242 | 222 | 206 | 189 | 107 | 96 | 110 | 118 | 129 |
| Thrombocyte count (x10^9/L) | 72 | 87 | 130 | 136 | 241 | 229 | 247 | 202 | 151 |

## Slide 2
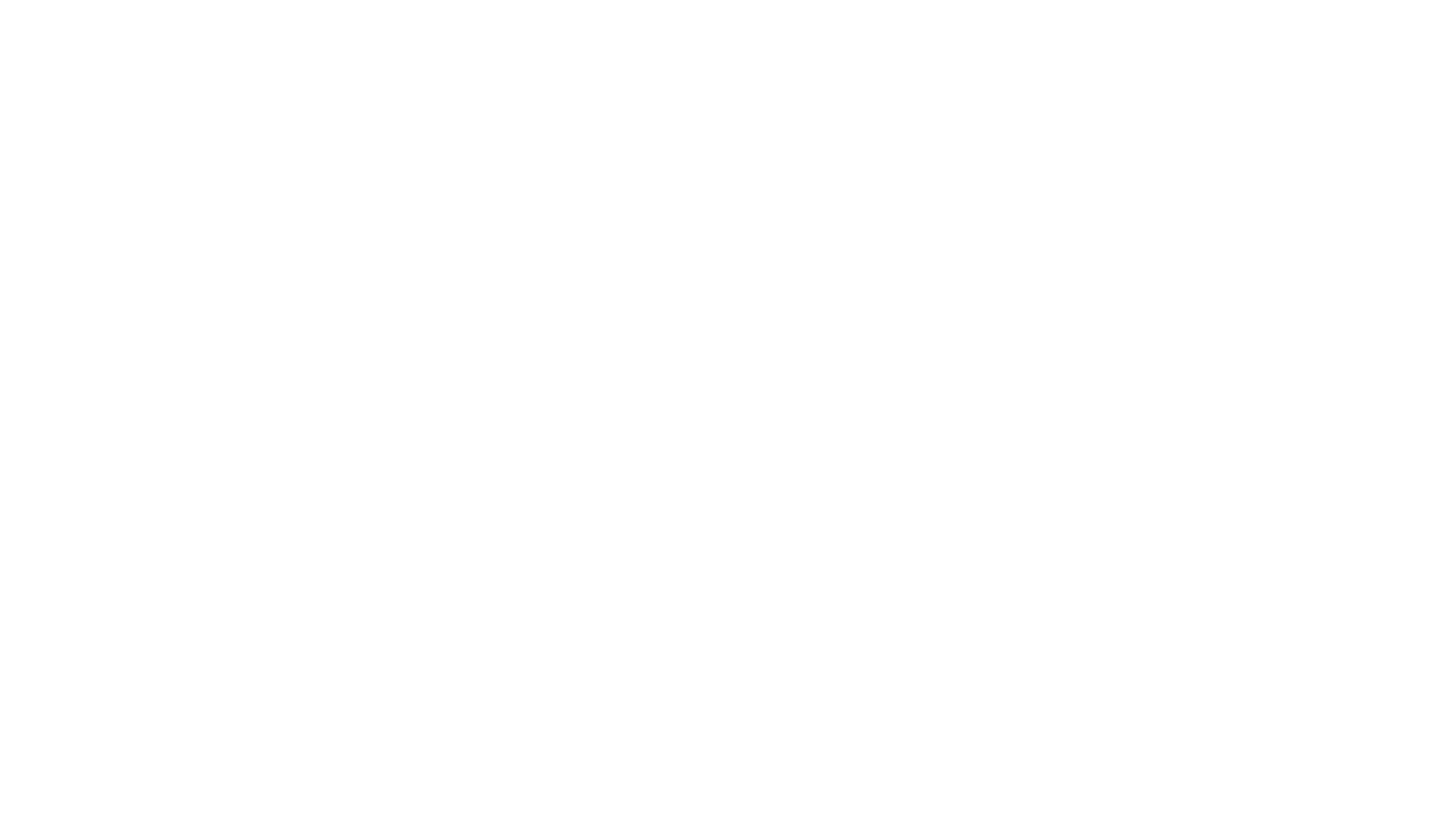

Supplement: Supplementary file 1 — Figure S1: Overview of biochemical evolution during follow‐up. (A) Graphical representation of hematocrit and thrombocyte count during follow up. Venesection was not performed on week 15 and 22. (B) Numeric values of hematocrit, hemoglobin concentration, and thrombocyte count during follow‐up. [file JHA2-4-1143-s001.pptx]
